# Towards proactive palliative care in oncology: developing an explainable EHR-based machine learning model for mortality risk prediction

**DOI:** 10.1186/s12904-024-01457-9

**Published:** 2024-05-20

**Authors:** Qingyuan Zhuang, Alwin Yaoxian Zhang, Ryan Shea Tan Ying Cong, Grace Meijuan Yang, Patricia Soek Hui Neo, Daniel SW Tan, Melvin LK Chua, Iain Beehuat Tan, Fuh Yong Wong, Marcus Eng Hock Ong, Sean Shao Wei Lam, Nan Liu

**Affiliations:** 1 Division of Supportive and Palliative Care, National Cancer Centre Singapore, 30 Hospital Blvd, Singapore, 168583 Singapore; 2 Division of Medical Oncology, National Cancer Centre Singapore, Singapore, Singapore; 3 Lien Centre of Palliative Care, Duke-NUS Medical School, Singapore, Singapore; 4 Division of Clinical Trials and Epidemiological Sciences, National Cancer Centre Singapore, Singapore, Singapore; 5 Data Computational Science Core, National Cancer Centre Singapore, Singapore, Singapore; 6 Division of Radiation Oncology, National Cancer Centre Singapore, Singapore, Singapore; 7 Department of Cancer Informatics, National Cancer Centre Singapore, Singapore, Singapore; 8 Health Services Research Centre, SingHealth, Singapore; 9 Centre for Quantitative Medicine, Duke-NUS Medical School, Singapore, Singapore; 10 Program in Health Services and Systems Research, Duke-NUS Medical School, Singapore, Singapore; 11 Saw Swee Hock School of Public Health, National University of Singapore, Singapore, Singapore

**Keywords:** Oncology, Palliative Medicine, Electronic Health Records, Machine learning, Clinical decision support systems

## Abstract

**Background:**

Ex-ante identification of the last year in life facilitates a proactive palliative approach. Machine learning models trained on electronic health records (EHR) demonstrate promising performance in cancer prognostication. However, gaps in literature include incomplete reporting of model performance, inadequate alignment of model formulation with implementation use-case, and insufficient explainability hindering trust and adoption in clinical settings. Hence, we aim to develop an explainable machine learning EHR-based model that prompts palliative care processes by predicting for 365-day mortality risk among patients with advanced cancer within an outpatient setting.

**Methods:**

Our cohort consisted of 5,926 adults diagnosed with Stage 3 or 4 solid organ cancer between July 1, 2017, and June 30, 2020 and receiving ambulatory cancer care within a tertiary center. The classification problem was modelled using Extreme Gradient Boosting (XGBoost) and aligned to our envisioned use-case: “Given a prediction point that corresponds to an outpatient cancer encounter, predict for mortality within 365-days from prediction point, using EHR data up to 365-days prior.” The model was trained with 75% of the dataset (*n* = 39,416 outpatient encounters) and validated on a 25% hold-out dataset (*n* = 13,122 outpatient encounters). To explain model outputs, we used Shapley Additive Explanations (SHAP) values. Clinical characteristics, laboratory tests and treatment data were used to train the model. Performance was evaluated using area under the receiver operating characteristic curve (AUROC) and area under the precision-recall curve (AUPRC), while model calibration was assessed using the Brier score.

**Results:**

In total, 17,149 of the 52,538 prediction points (32.6%) had a mortality event within the 365-day prediction window. The model demonstrated an AUROC of 0.861 (95% CI 0.856–0.867) and AUPRC of 0.771. The Brier score was 0.147, indicating slight overestimations of mortality risk. Explanatory diagrams utilizing SHAP values allowed visualization of feature impacts on predictions at both the global and individual levels.

**Conclusion:**

Our machine learning model demonstrated good discrimination and precision-recall in predicting 365-day mortality risk among individuals with advanced cancer. It has the potential to provide personalized mortality predictions and facilitate earlier integration of palliative care.

**Supplementary Information:**

The online version contains supplementary material available at 10.1186/s12904-024-01457-9.

## Background

In their last year of life, individuals with advanced cancer face costly and over-medicalized care, high unaddressed needs, and decreasing quality of life [1–9]. An early palliative care approach is essential to improve end-of-life outcomes, including symptom management, psychoeducation for patient and caregiver empowerment, and advance care planning [10–13]. Yet, many patients with advanced cancer in the real-world setting may either not receive palliative care, or receive it late into their disease trajectory [14–17]. Given workforce limitations, one proposed approach would be to use short-term mortality as a surrogate for identifying patients with high probability of palliative needs and most likely to benefit from palliative care [18–20]. 

Machine learning models trained on Electronic Health Record (EHR) data have shown promise in cancer prognostication, where advanced computational techniques are used to model linear and non-linear patterns within big datasets [19, 21]. The ability to leverage on routine data is attractive as it avoids burdensome external data entry and workflow disruptions. However, there remains several gaps within published literature.

First, while many published cancer prognostic models show promising discriminatory performance, the majority had high or uncertain risk of biasness, with incomplete reporting of modelling processes, and selective reporting of performance metrics [21, 22]. Specific to performance metrics among general oncology models, most models demonstrate low positive predictive value (0.45–0.53) and sensitivity (0.27–0.60), underperforming at the actual task of identifying patients who would die [21]. Second, alignment of model development strategy with articulated use-case is also critically missing in literature [23–26]. For example, some oncology prognostic models were developed on all-stage cancer cohorts despite the proposed use-case of increasing palliative care interventions. This fails to account that clinical implications and actions between early and advanced stage cancers can be vastly different when provided with a prediction of short-term mortality [27–29]. Third, if a model is designed for use as a clinical decision support system, reporting the model without intuitive explanations to model performance can negatively impact trust and adoption at clinical implementation [30]. In addition, complex models with automated feature selection and engineering may generate largely non-interpretable predictions [31]. 

This manuscript addresses gaps highlighted above. We aimed to develop and validate an explainable machine learning model trained on EHR data of advanced cancer patients, predicting for risk of 365-day mortality. Envisioning model output to nudge clinicians towards a palliative care approach, we aimed to enhance model interpretability by leveraging on prognostic literature and domain knowledge for feature engineering [32]. Systematic reporting of this study follows the Transparent Reporting of a Multivariable Prediction Model for Individual Prognosis or Diagnosis (TRIPOD) reporting guideline for prediction model development and validation [33]. 

## Methods

### Study design

We identified our cohort from patients with advanced cancer registered with the National Cancer Centre Singapore (NCCS). NCCS maintains a cancer-specific data repository with human-in-loop processes that registers cancer diagnosis and stage for each newly diagnosed patient. For each patient, data spanning 1st July 2016 to 31st December 2021 was extracted from the MOSAIQ Oncology Information System and SingHealth’s Enterprise Analytic Platform (eHints), which are unified data repositories that combine data from various healthcare transactional systems [34]. 

### Participants

Our cohort consisted of adults (age ≥ 18) diagnosed with Stage 3 or Stage 4 solid organ cancer between 1st July 2017 to 30th June 2020. To allow sufficient data for prediction, these patients were required to have at least two outpatient encounters within NCCS between 1st July 2017 to 31st December 2020. Non-residents were excluded from the cohort as their mortality outcome were not accurately reflected in local databases.

### Problem framing

**Fig. 1:**
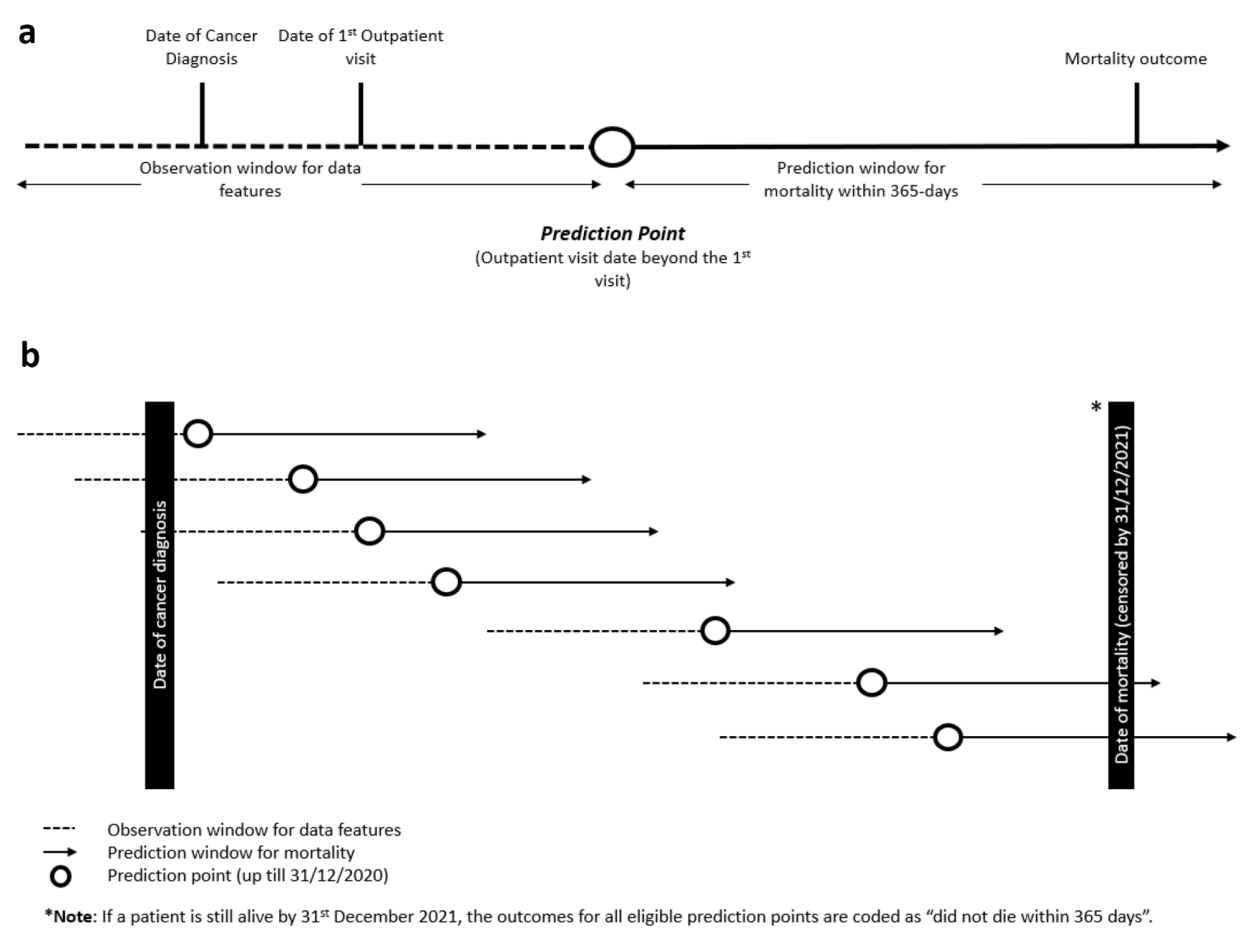
**a**: Framing of the risk prediction problem. **b**: Sliding window of prediction points along the timeline for a single patient

We framed our classification problem to match the use-case: “Given a prediction point that corresponds to an actualized outpatient cancer visit, predict for mortality within 365-days from prediction point, using EHR data up to 365-days prior.” *(*Fig. 1a*)* This prediction point effectively divides any patient’s EHR timeline into past events and a virtual future. To allow baseline data to be available for prediction, we restricted predictions to the 2nd outpatient visit and beyond. Patients were allowed more than one prediction point to capture their disease and treatment trajectory over time. To reduce over-training on samples with clustered visits, we only allowed one prediction point per month for those with more than the median number of outpatient visits (Fig. 1b).

### Outcome

Outcome was 365-day mortality from prediction point. Mortality date was obtained from the Singapore Registry of Births and Deaths and censored by 31st December 2021. Outcome was assumed complete as death registrations are mandatory by law.

### Data pre-processing

Oncologists, palliative specialists, and data scientists were involved in feature selection and engineering. Our data included 5 categories of data commonly available within EHR and clinically relevant to prognostication: (1) Demographics; (2) Clinical Characteristics; (3) Laboratory and Physical measurements; (4) Systemic cancer treatment; and (5) Healthcare visits. (Additional File 1: Table S1 and Table S2)

To derive features on systemic cancer treatment, we extracted dispensed drug data and mapped them to the World Health Organisation (WHO) Anatomical Therapeutic Chemical (ATC) classification. The WHO ATC classification is a system of alphanumeric codes developed for the classification of drugs in a hierarchy with five different levels. Subgroup L01 (with its subcodes) are antineoplastic agents, while subgroup L02 (with its subcodes) are cancer endocrine therapies [35]. We categorised cancer treatments under subgroups of “L01A, L01B, L01C, L01D, L01E, L01F, L01X, L02A, L02B, and Trial drugs [35]. Additionally, we generated cumulative counts of unique cancer drugs as a surrogate for change in cancer treatment line as this tends to portend poorer prognosis.

To derive comorbidities, we extracted International Classification of Diseases, Ninth and Tenth Revision (ICD-9 and ICD-10) diagnosis codes and transformed them into Elixhauser diagnosis categories using R package ‘comorbidity’ version 1.0.5 [36]. To represent laboratory test results and body mass index (BMI), we summarized data with minimum, maximum, median, standard deviation, and latest available reading [37]. Engineered features such as healthcare utilization count as well as elapsed time from diagnosis were computationally derived.

### Missing data handling

Longitudinal EHR data is often sparsely distributed, irregularly clustered, and incomplete [38]. Missingness within EHR data is “not missing at random” (NMAR) as the probability of missing data could be linked to disease severity, healthcare use, or a lack of clinical indication to collect the data [39]. Missingness is informative and should be incorporated within the modelling [40]. Boosted tree models such as XGBoost can handle missingness in features directly, as it is able to branch directions for missing values learned during training by itself (sparsity-aware split finding) [41]. Additional File 1: Table S2 provides a summary on missing data.

### Statistical analysis and modelling

We developed the boosted tree model on Python version 3.9.16 using XGBoost (xgboost version 1.7.5). The data was split with ratio of 75:25 data for training and validation. Area under the receiver operating characteristic curve (AUROC) was used as the primary performance metric, as it reflects trade-off between sensitivity and specificity. Because AUROC is misleadingly high in datasets with class imbalance, we reported the area under the precision-recall curve (AUPRC) as it measures trade-off between positive predictive value and sensitivity [42]. The calibration plot and Brier score were used to compare predicted vs. observed rates of 365-day mortality [43]. To explain model output, we used Shapley Additive Explanations (SHAP) values (shap 0.41.0), a model-agnostic methodology that improves transparency and interpretability of machine-learning models. SHAP values are based on a cooperative game theoretic approach, where contribution of each feature towards a prediction is calculated by comparing changes in the prediction, averaged across all possible combinations of input features [44, 45]. The model agnostic explainer used was TreeSHAP, which leverages on the structure of trees to approximate the Shapley values for each feature while providing feature attribution scores for predictions made by tree-based models [46]. 

## Results

A total of 5926 patients with 52,538 prediction points were included in this study. (Additional File 1: Figure S1) To prevent data leakage between training and validation sets, the 75 − 25 split was carried out at patient-level. The training cohort consisted of 39,416 prediction points among 4444 patients, while the test cohort consisted of 13,122 prediction points among 1482 patients.

The mean age of our population was 66.3 (Standard Deviation [SD] 11.5) years with 64.2% being male and majority (84.3%) of Chinese ethnicity. A total of 3725 patients (62.9%) had stage 4 cancer while 2201 patients (37.1%) had stage 3 cancer. By censor date of 31st December 2021, 3316 (55.6%) patients in the cohort had demised. (Table 1) In total, 17,149 of the 52,538 prediction points (32.6%) had a mortality event within the 365-day prediction window.

**Table 1:** Study population characteristics (*n* = 5926)

Variable	Number (%)
Age at Diagnosis	
Mean (SD)	66.3 (11.5)
Median (IQR)	67 (15)
Female	2122 (35.8)
Race	
Chinese	4996 (84.3)
Malay	506 (8.5)
Indian	243 (4.1)
Others	181 (3.1)
Cancer Stage	
III	2201 (37.1)
IV	3725 (62.9)
Median number of comorbidities (IQR)	3 (2)
ICD-10^1^ Cancer diagnosis	
C00-C14 Malignant neoplasms of lip, oral cavity, and pharynx	508 (8.6)
C15-C21 Malignant neoplasms of gastrointestinal tract	1620 (27.3)
C22-C26 Malignant neoplasms of hepatobiliary system	668 (11.3)
C30-C39 Malignant neoplasms of respiratory and intrathoracic organs	1353 (22.8)
C40-C41 Malignant neoplasms of bone and articular cartilage	4 (0.1)
C43-C44 Melanoma and other malignant neoplasms of skin	24 (0.4)
C45-C49 Malignant neoplasms of mesothelial and soft tissue	42 (0.7)
C50-C50 Malignant neoplasms of breast	465 (7.8)
C51-C58 Malignant neoplasms of female genital organs	226 (3.8)
C60-C63 Malignant neoplasms of male genital organs	703 (11.9)
C64-C68 Malignant neoplasms of urinary tract	226 (3.8)
C73-C75 Malignant neoplasms of thyroid and other endocrine glands	26 (0.4)
C76-C80 Malignant neoplasms of ill-defined, other secondary and unspecified sites	99 (1.7)
Mortality by 31st December 2021	3316 (55.6)

^1^ICD10: International Classification of Diseases 10th Revision

### Model

Model performance metrics on the validation cohort are reported in Table 2. The confusion matrix and model parameters can be found in the Additional file (Table S3 and S4 respectively). Set at a default classification threshold of 0.5, our model achieved an Accuracy of 0.781 (95% CI 0.774–0.788), AUROC of 0.861 (95% CI 0.856–0.867) and AUPRC of 0.771. In terms of model calibration, the Brier score was 0.147 with slight overestimations of 365-day mortality risk (calibration plot shown in Additional file 1: Figure S2).

**Table 2:** Performance metrics of XGBoost Model

Accuracy (95% CI)	0.781 (0.774–0.788)
Precision	0.660
Recall / Sensitivity	0.734
Specificity	0.805
F1^1^	0.695
Brier score	0.147
AUROC^2^ (95% CI)	0.861 (0.856–0.867)
AUPRC^3^	0.771

^1^F1: Harmonic mean of precision and recall, ^2^ROC-AUC: Area under the Curve of Receiver Operating Characteristics, ^3^PR-AUC: Area under the Curve of Precision-Recall Curve

### Explainability

**Fig. 2:**
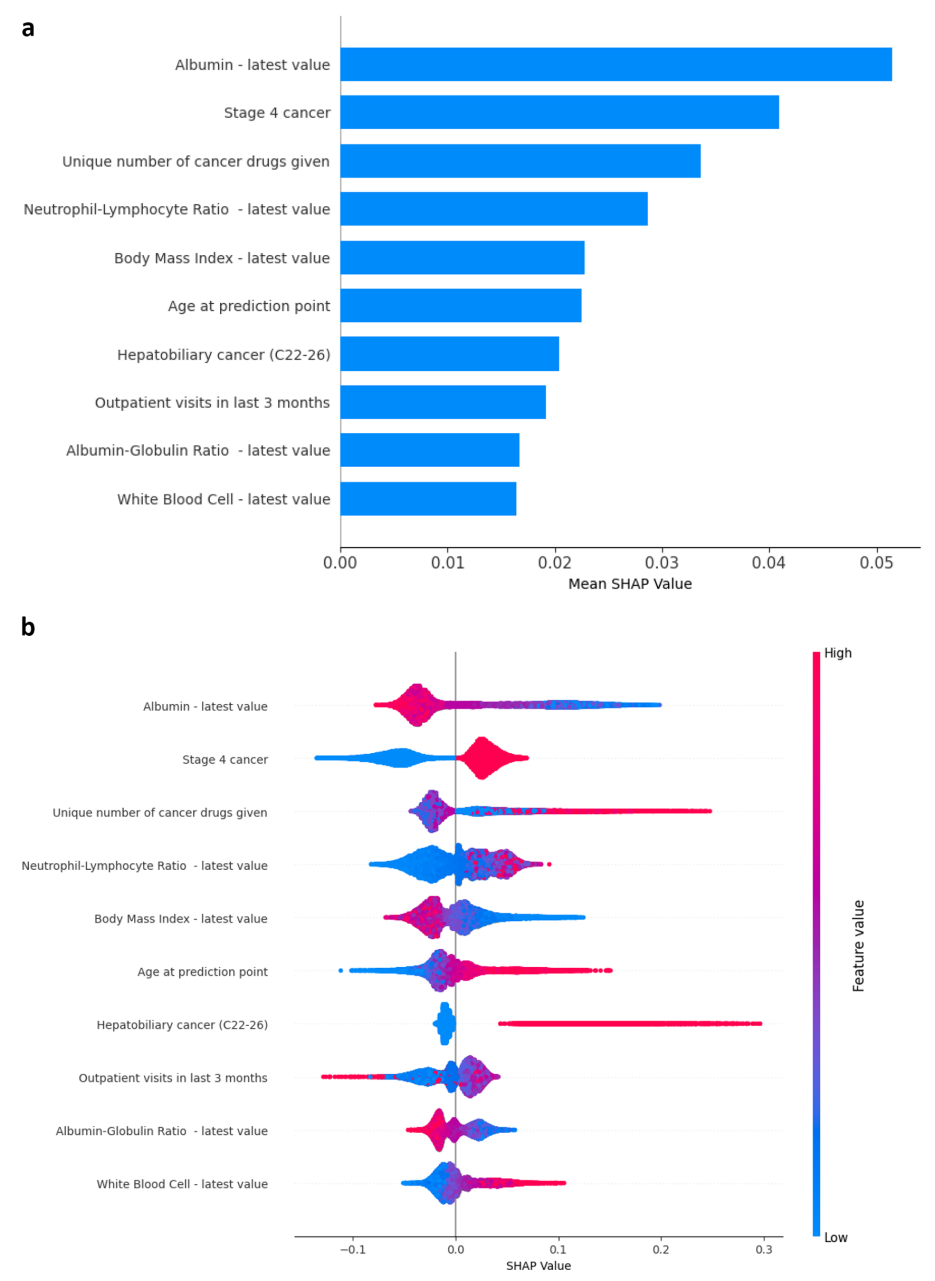
**a**. Bar summary of top 10 data features within the model. **b**. Feature plot summary of top 10 data features within the model

Figure 2a provides a summary ranking of the topmost data features (from highest to lowest SHAP values) within the model. The model itself considers all features and SHAP values can be calculated for all features. However, we show only the top 10 features for the sake of brevity. The top 3 impactful data features are the latest albumin value, stage 4 cancer on diagnosis, and unique number of cancer drugs given.

Figure 2b shows the interaction between value of each feature and its impact on model prediction. Similarly, we illustrate the top 15 features. The values for numeric features are normalized and represented along a colour gradient with red for larger value and blue for smaller value of the feature. The values for categorical features are similarly represented with red for present (value = 1.00) and blue for absent (value = 0.00). Within each feature, the line is then visualized by plotting individual-coloured dots that represents each prediction along its SHAP value (x-axis). A negative SHAP value (extending to the left) indicates a reduced probability for mortality while a positive SHAP value (extending to the right) indicates an increased probability of mortality. For example, we find that the lower the albumin value, the higher the probability for mortality (the y-axis line extending to the left is mostly red while the line extending to the right turns increasingly blue). Predictions with stage 4 cancer are associated with a higher probability for mortality, as they cluster towards the right side of the y-axis line.

## Discussion

In this study, we trained and validated an XGBoost model using structured EHR data of advanced cancer patients. The model performed with excellent discrimination (AUROC 0.861), precision-recall (AUPRC 0.771), and accuracy (0.781) in predicting for the last year of life. Comparing against most similar published machine learning models in general cancer cohorts, we report a similar AUROC (0.812–0.890) and much higher AUPRC (0.340–0.462) [27, 28, 47, 48]. A high precision-recall is important to identify the few patients that will die within a year without overestimating the risk of death for the majority of patients who will actually survive, especially within resource-limited settings [42]. 

From the outset, we framed this AI development as a clinician decision support tool where predictions of high-risk mortality within 365-days may nudge clinicians towards considering involvement of palliative care, earlier anticipatory care discussions, as well as re-assessing the risk-benefit ratios of standard-of-care next line therapies. Hence, model interpretability is essential for user adoption and acceptance [49]. Eschewing the practice of a completely data-driven approach to feature development, we instead leveraged on domain knowledge of oncologists and palliative specialists in feature design to help with subsequent interpretability [32, 50]. For example, we recognise that disease control rates drop and risk of disease mortality increases with change in lines of cancer treatment [51]. Hence, an engineered feature of cumulative counts of unique cancer drug as a surrogate for cancer treatment line change was added, which became the third most important feature within our XGBoost model (Fig. 2a). Another example is where we incorporated strong literature evidence that elevated Neutrophil-Lymphocyte ratio (NLR) is associated with poor prognosis, and engineered features around NLR instead of providing raw neutrophil and lymphocyte data to the model [52]. This feature is the fourth most important *(*Fig. 2b*)* where higher NLR values are associated with increased probability for mortality. Our approach of developing explainable models with engineered features that comport with literature and clinical knowledge resonates with the clinician’s own intuitive understanding of prognostication and may increase model adoption [53]. 

**Fig. 3:**
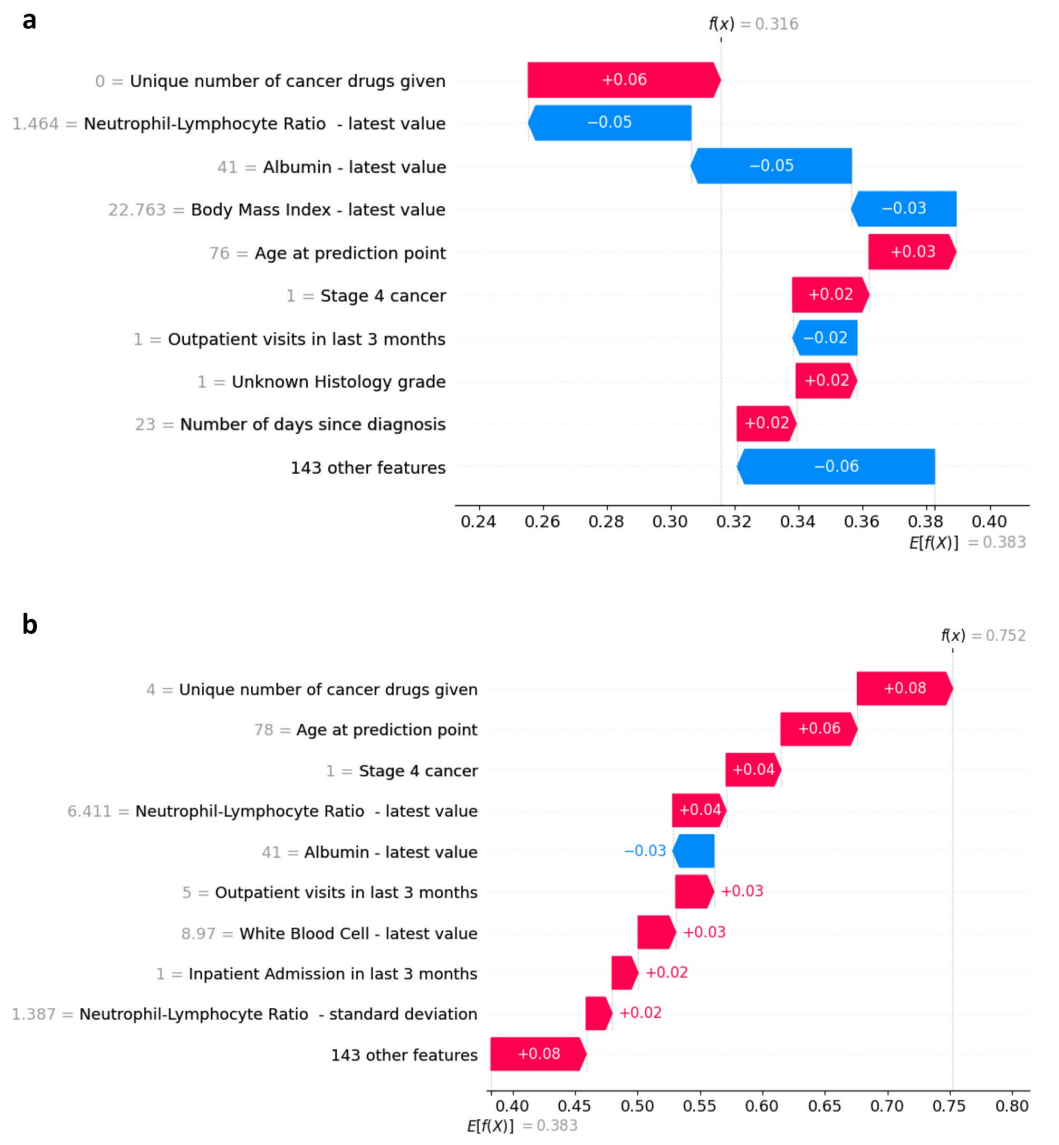
**a**. Individualized prediction for a true-negative case. **b**. Individualized prediction for a true-positive case

Beyond global interpretability for a “black-box” machine-learning model, we have taken a next step by providing individual prediction explanations. Commonly, a binary classification model requires set probability threshold (set at 0.5 in our model), yet a patient with predicted probability of 0.49 may not be necessarily different in terms of risk from a patient with predicted probability of 0.51. Instead of using binary mortality prediction as a strict rule, we feel that visualizing predicted probabilities with model explainers will provide better clinical decision support for further clinical evaluation and interventions. Figure 3a and b shows the composition of individualized predictions for a 76-year-old Chinese gentleman with T3N0M1 lung cancer and comorbidities of hypertension and diabetes. “**E[fX]]**” denotes the average predicted probability of 365-day mortality for our entire cohort without considering any data features. “**f(x)**” denotes the final predicted probability of 365-day mortality after summing up all the feature contributions. Read from the bottom up, each data feature either increases (red arrows) or decreases (blue arrows) the probability of 365-day mortality additively. In Fig. 3a, this prediction was done 23 days post diagnosis, where among other features, he had normal albumin (41.0 g/dl), low neutrophil-lymphocyte ratio (1.46) and healthy body mass index (22.8). The model predicted patient to have a 31.6% risk of dying in the next 365-days, which turned out to be a true-negative prediction. In Fig. 3b, this prediction was done 505 days post diagnosis on the same patient, where patient’s albumin remained normal (41.0 g/dl), but being older, having received 4 different anti-cancer drugs, and having a higher neutrophil-lymphocyte ratio (6.41) increased his probability of mortality. The model predicted patient to have an 75.2% risk of dying in the next 365-days, which turned out to be a true-positive prediction.

Our model shows potential for clinical implementation in the cancer outpatient setting. The model output can be used in several ways. First, regular reports on identified outpatients can be provided to a back-end triage and case-management system. By proactively reaching out to these at-risk patients and offering regular palliative needs screening, issues can be identified and managed promptly. Second, model explanations and prompts can be sent to oncologists to increase their prognostic awareness, nudge them towards early anticipatory care planning, and reassess the risk-benefit ratios of next-line therapies. Third, the ability to identify the ex-ante end-of-life cancer cohort aids targeted study, formulation of healthcare policy, and prospective outcomes tracking around this at-risk group.

This study has several limitations. First, the model was trained and validated within a single centre advanced cancer cohort, and external validation will be needed to determine generalizability. Second, because cancer treatment continues to evolve rapidly, temporal validation is needed to determine performance drift over time. Third, algorithmic fairness will also need to be ascertained in subsequent work by validating performance within key demographic subgroups (e.g. age groups, ethnicity, and gender) [54]. Fourth, our model was trained on advanced cancer patients on diagnosis and does not include patients with early staged cancers on diagnosis with subsequent metastatic relapse. Identification of metastatic relapse is lacking even in established cancer registries like the Surveillance, Epidemiology, and End Results (SEER) Cancer database, and this is a problem that needs to be solved before any model can be used for patients with metastatic relapse [55]. Fifth, the model relies on processed EHR data obtained from institutional data repositories. Future model deployment will require access to these same data repositories and platforms, instead of direct implementation within the operational EHR environment. Lastly, as an AI tool for clinical decision support, performance metrics itself may not translate to real-world results, if clinicians do not act on the prediction, or resource limitations reduces the number of at-risk patients who can receive interventions. With recent national focus on end-of-life care within population health, we envision that palliative capacity and capabilities will be bolstered to meet the needs of these additionally identified patients [56]. In addition, we are exploring in-silico net-benefit analysis to study impact of the model on clinical outcomes based on simulated scenarios [57]. 

## Conclusions

We have developed a prognostic tree-based model using structured EHR data, which possesses satisfactory discrimination and precision-recall capabilities. Our model development approach places emphasis on problem framing, feature hand crafting using domain expertise, and interpretable outputs aimed at both global and individual level prediction. While the model performance provides sufficient evidence in its use-case, further external validation is needed to confirm its robustness for real-world implementation. Further work is planned to conduct a prospective multi-centre validation study to simulate our envisioned use-case by handling actual data volumes of cancer outpatients weekly, allowing us to ascertain the model’s operability and efficiency within a real-world situation. Ultimately, this will enable us to refine and validate an AI solution that enables systematic ex-ante identification of cancer patients at-risk of mortality, with proactive palliative interventions triggered for the said individual.

### Electronic supplementary material

Below is the link to the electronic supplementary material.

Supplementary Material 1

## Data Availability

The datasets used and/or analysed during the current study are available from the corresponding author on reasonable request. The data pre-processing and model development codes are available on https://github.com/SHS-HSRC/PROTECH-Study.

## Declarations

### Concept and design

Qingyuan Zhuang, Alwin Zhang, Grace Yang, Patricia Neo, Daniel Tan, Fuh Yong Wong, Marcus Ong, Sean Lam, Liu Nan.

## References

[1] Luta X, Diernberger K, Bowden J, Droney J, Hall P, Marti J (2022). Intensity of care in cancer patients in the last year of life: a retrospective data linkage study. Br J Cancer.

[2] Mariotto AB, Enewold L, Zhao J, Zeruto CA, Yabroff KR (2020). Medical Care costs Associated with Cancer Survivorship in the United States. Cancer Epidemiol Biomarkers Prev.

[3] Goldsbury DE, Yap S, Weber MF, Veerman L, Rankin N, Banks E (2018). Health services costs for cancer care in Australia: estimates from the 45 and up study. PLoS ONE.

[4] Luengo-Fernandez R, Leal J, Gray A, Sullivan R (2013). Economic burden of cancer across the European Union: a population-based cost analysis. Lancet Oncol.

[5] Zhuang Q, Chong PH, Ong WS, Yeo ZZ, Foo CQZ, Yap SY (2022). Longitudinal patterns and predictors of healthcare utilization among cancer patients on home-based palliative care in Singapore: a group-based multi-trajectory analysis. BMC Med.

[6] Lee J, Shafiq M, Malhotra R, Ozdemir S, Teo I, Malhotra C (2022). Trajectories of Health-related quality of life in patients with Advanced Cancer during the Last Year of Life: findings from the COMPASS study. BMC Palliat Care.

[7] Chua GP, Pang GSY, Yee ACP, Neo PSH, Zhou S, Lim C (2020). Supporting the patients with advanced cancer and their family caregivers: what are their palliative care needs?. BMC Cancer.

[8] Giesinger JM, Wintner LM, Oberguggenberger AS, Gamper EM, Fiegl M, Denz H (2011). Quality of life trajectory in patients with Advanced Cancer during the Last Year of Life. J Palliat Med.

[9] Seow H, Barbera L, Sutradhar R, Howell D, Dudgeon D, Atzema C (2011). Trajectory of performance status and symptom scores for patients with cancer during the last six months of life. J Clin Oncol.

[10] Rimmer B, Crowe L, Todd A, Sharp L (2022). Assessing unmet needs in advanced cancer patients: a systematic review of the development, content, and quality of available instruments. J Cancer Surviv.

[11] Bakitas MA, Tosteson TD, Li Z, Lyons KD, Hull JG, Li Z (2015). Early Versus delayed initiation of Concurrent Palliative Oncology Care: patient outcomes in the ENABLE III Randomized Controlled Trial. J Clin Oncol.

[12] Bernacki R, Paladino J, Neville BA, Hutchings M, Kavanagh J, Geerse OP (2019). Effect of the Serious Illness Care Program in Outpatient Oncology: a Cluster Randomized Clinical Trial. JAMA Intern Med.

[13] Bestvina CM, Polite BN (2017). Implementation of Advance Care Planning in Oncology: a review of the literature. JOP.

[14] Janah A, Gauthier LR, Morin L, Bousquet PJ, Le Bihan C, Tuppin P (2019). Access to palliative care for cancer patients between diagnosis and death: a national cohort study. Clin Epidemiol.

[15] Hui D, Elsayem A, De La Cruz M, Berger A, Zhukovsky DS, Palla S (2010). Availability and Integration of Palliative Care at United States Cancer centers. JAMA.

[16] Knaul FM, Farmer PE, Krakauer EL, Lima LD, Bhadelia A, Kwete XJ (2018). Alleviating the access abyss in palliative care and pain relief—an imperative of universal health coverage: the Lancet Commission report. Lancet.

[17] Bennett MI, Ziegler L, Allsop M, Daniel S, Hurlow A (2016). What determines duration of palliative care before death for patients with advanced disease? A retrospective cohort study of community and hospital palliative care provision in a large UK city. BMJ Open.

[18] Avati A, Jung K, Harman S, Downing L, Ng A, Shah NH. Improving palliative care with deep learning. BMC Med Inform Decis Mak [Internet]. 2018 Dec 12 [cited 2020 Aug 19];18(Suppl 4). https://www.ncbi.nlm.nih.gov/pmc/articles/PMC6290509/. 10.1186/s12911-018-0677-8 PMC6290509 30537977

[19] Downar J, Wegier P, Tanuseputro P (2019). Early identification of people who would benefit from a Palliative Approach—moving from Surprise to Routine. JAMA Netw Open.

[20] Hui D, Paiva CE, Del Fabbro EG, Steer C, Naberhuis J, van de Wetering M (2019). Prognostication in Advanced Cancer: update and directions for Future Research. Support Care Cancer.

[21] Lu SC, Xu C, Nguyen CH, Geng Y, Pfob A, Sidey-Gibbons C (2022). Machine learning-based short-term mortality prediction models for patients with Cancer using Electronic Health Record Data: systematic review and critical Appraisal. JMIR Med Inf.

[22] Dhiman P, Ma J, Andaur Navarro CL, Speich B, Bullock G, Damen JAA (2022). Methodological conduct of prognostic prediction models developed using machine learning in oncology: a systematic review. BMC Med Res Methodol.

[23] Seneviratne MG, Shah NH, Chu L. Bridging the implementation gap of machine learning in healthcare. BMJ Innovations [Internet]. 2020 Apr 1 [cited 2023 Feb 15];6(2). https://innovations.bmj.com/content/6/2/45.

[24] Jung K, Kashyap S, Avati A, Harman S, Shaw H, Li R (2021). A framework for making predictive models useful in practice. J Am Med Inform Assoc.

[25] Li RC, Asch SM, Shah NH (2020). Developing a delivery science for artificial intelligence in healthcare. Npj Digit Med.

[26] Lauritsen SM, Thiesson B, Jørgensen MJ, Riis AH, Espelund US, Weile JB (2021). The Framing of machine learning risk prediction models illustrated by evaluation of sepsis in general wards. NPJ Digit Med.

[27] Parikh RB, Manz C, Chivers C, Regli SH, Braun J, Draugelis ME et al. Machine Learning Approaches to Predict 6-Month Mortality Among Patients With Cancer. JAMA Netw Open [Internet]. 2019 Oct 25 [cited 2020 Oct 28];2(10). https://www.ncbi.nlm.nih.gov/pmc/articles/PMC6822091/. 10.1001/jamanetworkopen.2019.15997 PMC6822091 31651973

[28] Manz CR, Parikh RB, Small DS, Evans CN, Chivers C, Regli SH et al. Effect of integrating machine learning mortality estimates with behavioral nudges to clinicians on Serious Illness conversations among patients with Cancer: a stepped-Wedge Cluster Randomized Clinical Trial. JAMA Oncol. 2020;e204759. 10.1001/jamaoncol.2020.4759 PMC7563672 33057696

[29] Elfiky AA, Pany MJ, Parikh RB, Obermeyer Z (2018). Development and application of a machine Learning Approach to assess short-term mortality risk among patients with Cancer starting chemotherapy. JAMA Netw Open.

[30] Char DS, Shah NH, Magnus D (2018). Implementing Machine Learning in Health Care - addressing ethical challenges. N Engl J Med.

[31] Gosiewska A, Kozak A, Biecek P (2021). Simpler is better: lifting interpretability-performance trade-off via automated feature engineering. Decis Support Syst.

[32] Deng C, Ji X, Rainey C, Zhang J, Lu W (2020). Integrating machine learning with human knowledge. iScience.

[33] Collins GS, Reitsma JB, Altman DG, Moons KGM (2015). Transparent reporting of a multivariable prediction model for individual prognosis or diagnosis (TRIPOD): the TRIPOD statement. Ann Intern Med.

[34] Electronic Health Intelligence System [Internet]. [cited 2020 Jul 12]. https://www.ihis.com.sg/Project_Showcase/Healthcare_Systems/Pages/eHINTS.aspx.

[35] WHO Collaborating Centre for Drug Statistics Methodology [Internet]. [cited 2022 Mar 26]. https://www.whocc.no/atc/structure_and_principles/.

[36] Elixhauser A, Steiner C, Harris DR, Coffey RM (1998). Comorbidity measures for use with administrative data. Med Care.

[37] Cascarano A, Mur-Petit J, Hernández-González J, Camacho M, de Toro Eadie N, Gkontra P (2023). Machine and deep learning for longitudinal biomedical data: a review of methods and applications. Artif Intell Rev.

[38] Hripcsak G, Albers DJ (2013). Next-generation phenotyping of electronic health records. J Am Med Inf Assoc.

[39] Wells BJ, Chagin KM, Nowacki AS, Kattan MW. EGEMS (Wash DC). 2013;1(3):1035. Strategies for Handling Missing Data in Electronic Health Record Derived Data. 10.13063/2327-9214.1035 PMC4371484 25848578

[40] Groenwold RHH (2020). Informative missingness in electronic health record systems: the curse of knowing. Diagn Prognostic Res.

[41] Chen T, Guestrin C, XGBoost:. A Scalable Tree Boosting System. In: Proceedings of the 22nd ACM SIGKDD International Conference on Knowledge Discovery and Data Mining [Internet]. New York, NY, USA: Association for Computing Machinery; 2016 [cited 2023 Apr 10]. pp. 785–94. (KDD ’16). 10.1145/2939672.2939785.

[42] Saito T, Rehmsmeier M (2015). The Precision-Recall plot is more informative than the ROC plot when evaluating Binary classifiers on Imbalanced datasets. PLoS ONE.

[43] Huang Y, Li W, Macheret F, Gabriel RA, Ohno-Machado L (2020). A tutorial on calibration measurements and calibration models for clinical prediction models. J Am Med Inform Assoc.

[44] Lundberg S, Lee SI. A Unified Approach to Interpreting Model Predictions [Internet]. arXiv; 2017 [cited 2023 Jan 20]. http://arxiv.org/abs/1705.07874.

[45] Lundberg SM, Erion G, Chen H, DeGrave A, Prutkin JM, Nair B (2020). From local explanations to Global understanding with explainable AI for trees. Nat Mach Intell.

[46] Lundberg SM, Erion GG, Lee SI. Consistent Individualized Feature Attribution for Tree Ensembles [Internet]. arXiv; 2019 [cited 2023 Feb 13]. http://arxiv.org/abs/1802.03888.

[47] Zachariah FJ, Rossi LA, Roberts LM, Bosserman LD (2022). Prospective Comparison of Medical oncologists and a machine learning model to Predict 3-Month Mortality in patients with metastatic solid tumors. JAMA Netw Open.

[48] Gensheimer MF, Aggarwal S, Benson KRK, Carter JN, Henry AS, Wood DJ (2020). Automated model versus treating physician for predicting survival time of patients with metastatic cancer. J Am Med Inf Assoc.

[49] Elshawi R, Al-Mallah MH, Sakr S (2019). On the interpretability of machine learning-based model for predicting hypertension. BMC Med Inf Decis Mak.

[50] Correia AHC, Lecue F. Human-in-the-Loop Feature Selection. Proceedings of the AAAI Conference on Artificial Intelligence. 2019;33(01):2438–45.

[51] Hanker LC, Loibl S, Burchardi N, Pfisterer J, Meier W, Pujade-Lauraine E (2012). The impact of second to sixth line therapy on survival of relapsed ovarian cancer after primary taxane/platinum-based therapy. Ann Oncol.

[52] Cupp MA, Cariolou M, Tzoulaki I, Aune D, Evangelou E, Berlanga-Taylor AJ (2020). Neutrophil to lymphocyte ratio and cancer prognosis: an umbrella review of systematic reviews and meta-analyses of observational studies. BMC Med.

[53] Watson J, Hutyra CA, Clancy SM, Chandiramani A, Bedoya A, Ilangovan K (2020). Overcoming barriers to the adoption and implementation of predictive modeling and machine learning in clinical care: what can we learn from US academic medical centers?. JAMIA Open.

[54] Xu J, Xiao Y, Wang WH, Ning Y, Shenkman EA, Bian J et al. Algorithmic fairness in computational medicine. eBioMedicine [Internet]. 2022 Oct 1 [cited 2023 Feb 23];84. https://www.thelancet.com/journals/ebiom/article/PIIS2352-3964(22)00432-7/fulltext. 10.1016/j.ebiom.2022.104250 PMC9463525 36084616

[55] Warren JL, Yabroff KR (2015). Challenges and opportunities in measuring cancer recurrence in the United States. J Natl Cancer Inst.

[56] SPEECH BY MINISTER FOR HEALTH, MR ONG YE KUNG, AT THE MOH WORK PLAN SEMINAR. 2022, 2 JUNE 2022 [Internet]. 2022 [cited 2023 Jan 13]. https://www.moh.gov.sg/news-highlights/details/speech-by-minister-for-health-mr-ong-ye-kung-at-the-moh-work-plan-seminar-2022-2-june-2022.

[57] Vickers AJ, Calster BV, Steyerberg EW (2016). Net benefit approaches to the evaluation of prediction models, molecular markers, and diagnostic tests. BMJ.

