# Supplementary material for: Towards proactive palliative care in oncology: developing an explainable EHR-based machine learning model for mortality risk prediction
Source: BMC Palliat Care. 2024 May 20;23:124. doi: 10.1186/s12904-024-01457-9 (PMC11103848; doi:10.1186/s12904-024-01457-9)
Supplement: Supplementary file 1 — Supplementary Material 1 [file 12904_2024_1457_MOESM1_ESM.docx]

**Additional File**

**Figure S1.** Flow diagram of the study participants

**7266** Stage 3 or Stage 4 solid organ cancer patients diagnosed between 1^st^ July 2017 to 30^th^ June 2020

**1340** did not meet inclusion criteria:

- 1300 patients who were non-residents.
- 20 patients with only 1 outpatient visit.
- 20 patients < 18 years old.

**5926** unique patients

**103055** *prediction points* between 1^st^ July 2017 to 31^st^ December 2020

Down-sampling of *prediction points* by allowing maximum of 1 *prediction point* per month.

**5926** unique patients

**52538** *prediction points* between 1^st^ July 2017 to 31^st^ December 2020

**Training Set**

**4444** unique patients

**39416** *prediction points* between 1^st^ July 2017 to 31^st^ December 2020

**Validation Set**

**1482** unique patients

**13122** *prediction points*

**Figure S2.** Calibration plot of the XGBoost model.


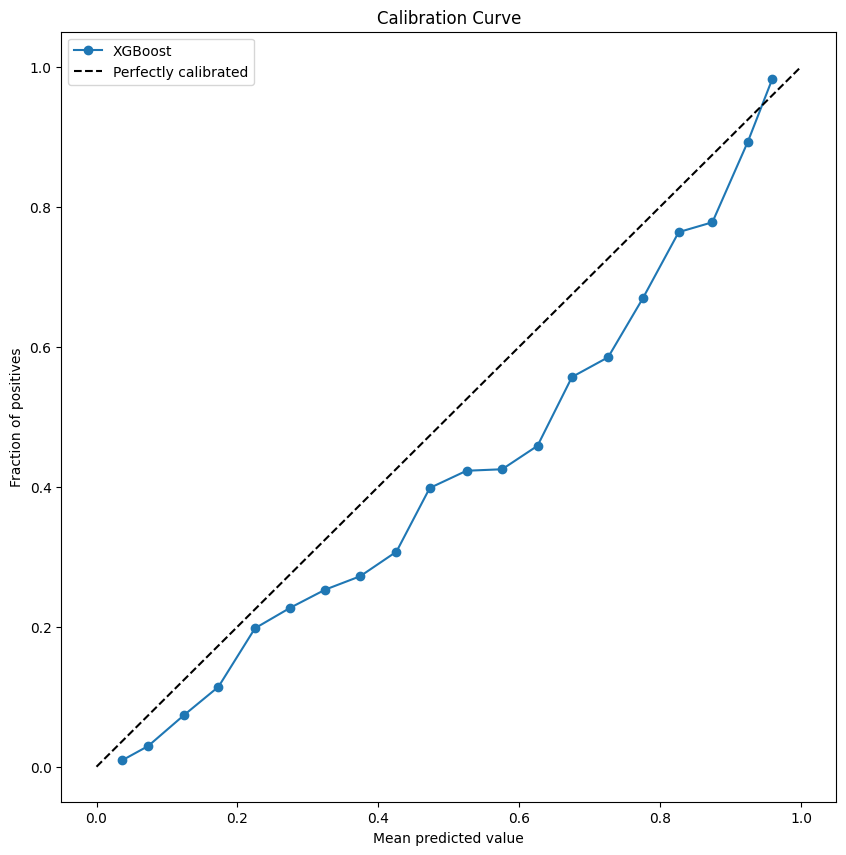


**Table S1.** **Extracted features from the Electronic Health Records**.

| **Category** | **Feature** | **Description** |
| --- | --- | --- |
| **Demographics** | Gender | Male or Female |
|  | Race | Chinese, Malay, Indian, Others |
|  | Age at visit | Age at *prediction point* |
| **Clinical characteristics** | Stage | Cancer stage 3 or 4 |
|  | ICD10 Diagnosis^1^ | ICD10 Diagnosis codes |
|  | TNM staging | Tumor (T), node (N), and metastasis (M) categories |
|  | Histological tumor grade | Grade 1, Grade 2, Grade 3, Grade 4, Grade 9 (undetermined) |
|  | Comorbidities | 31 Elixhauser^2^ comorbidities |
| **Lab & Physical measurements** | Albumin | Aggregated readings 365 days before *prediction point* (e.g. max, min, median, mean, standard deviation, latest reading) |
|  | Bilirubin |  |
|  | Creatinine clearance |  |
|  | Haemoglobin |  |
|  | Platelet count |  |
|  | White Blood Count |  |
|  | Neutrophil-Lymphocyte Ratio |  |
|  | Albumin-Globulin Ratio |  |
|  | Body Mass Index (BMI) |  |
| **Cancer drugs** | WHO ATC Codes^3^:   - L01 A-F, X - L02 A & B - Trial Drugs | Cancer drugs that are dispensed from date of diagnosis till *prediction point* |
|  | Count of unique drugs |  |
| **Healthcare Visits** | Count of Inpatient Visits | Cumulative number of visits 365, 180 or 30 days before *prediction point* |
|  | Count of Emergency Department Visits |  |
|  | Count of Outpatient Visits |  |
| **Other Feature Engineered variables** | Difference in days | Difference in days between diagnosis date and *prediction point* |

^1^ICD10: International Classification of Diseases 10^th^ Revision, ^2^Elixhauser comorbidities: A method of categorizing comorbidities of patients based on ICD diagnosis codes found in administrative data, ^3^WHO ATC Codes: World Health Organization Anatomical Therapeutic Chemical Codes

**Table S2. Summary statistics.**

Based on 52538 rows of patient data at the point of outpatient visit.

| Variable Name | Description | Values | No. of missing values |
| --- | --- | --- | --- |
| Number of days since diagnosis | Difference in days between diagnosis date and *prediction date* (days) | Min-Max: 0-1273  Mean, SD: 298, 256  Median, IQR: 216, 330 | 0 |
| Gender | Gender of the patient | Male: 32757 Female: 19781 | 0 |
| Race | Ethnic group of the patient | Chinese: 44639 Malay: 4198 Indian: 2167  Others: 1534 | 0 |
| Histology Grade | The grade categorises the appearance and aggressiveness of cancer cells | Grade 1: 1005 Grade 2: 13884  Grade 3: 10156  Grade 4: 5939  Grade 9: 21554 | 0 |
| Clinical T | T refers to the size and extent of the main tumor. The N refers to the number of nearby lymph nodes that have cancer. The M refers to whether the cancer has metastasized.  Clinical staging determines how much cancer there is based on physical/radiological examination. Pathological staging is determined after surgical removal of a tumor. | T0: 206  T1: 2155  T2: 4271  T3: 7817  T4: 7587  Tis: 15  TX: 13410 | 17077 |
| Clinical N |  | N0: 6002  N1: 6087  N2: 5954  N3: 4310  NX: 13108 | 17077 |
| Pathologic T |  | T0: 541  T1: 1415  T2: 3072  T3: 7977  T4: 5305  TX: 326 | 33902 |
| Pathologic N |  | N0: 6002  N1: 6087  N2: 5954  N3: 4310  NX: 13108 | 33892 |
| Lip, oral cavity and pharynx cancer (C00-C14) | Cancer Diagnosis in ICD-10 codes. This is human-curated within a cancer-specific data repository. | Count: 5168 | 0 |
| Digestive organ cancer (C15-C21) |  | Count: 14744 | 0 |
| Hepatobiliary cancer (C22-26) |  | Count: 4532 | 0 |
| Respiratory and intrathoracic organ cancer (C30-C39) |  | Count: 11818 | 0 |
| Bone and articular cartilage cancer (C40-C41) |  | Count: 50 | 0 |
| Melanoma or skin cancer (C43-C44) |  | Count: 279 | 0 |
| Mesothelial and soft tissue cancer (C45-C49) |  | Count: 393 | 0 |
| Breast cancer (C50) |  | Count: 5633 | 0 |
| Female genital organ cancer (C51-C58) |  | Count: 2068 | 0 |
| Male genital organ cancer (C60-C63) |  | Count: 5878 | 0 |
| Urinary tract cancer (C64-C68) |  | Count: 1834 | 0 |
| Cancer of thyroid and other endocrine glands (C73-C75) |  | Count: 130 | 0 |
| Ill-defined cancer, other secondary and unspecified sites (C76-C80) |  | Count: 403 | 0 |
| Cancer stage | Cancer Staging | Stage 3: 20499  Stage 4: 32039 | 0 |
| Albumin - median | Protein made by the liver which measures the liver and kidney function. It is generally applied to assess the nutritional status and has also been found to be an independent predictor of cancer survival.  The data is aggregated 1 year prior to each visit date. Each acronym denotes the following:   - med – Median value - mean – Mean - max – Maximum value - min – Minimum value - sd – Standard deviation - lat – Latest value | Min-Max: 15 - 51  Mean, SD: 37.6, 4.5 Median, IQR: 38.5, 6 | 6355 |
| Albumin - mean |  | Min-Max: 16.7 - 51  Mean, SD: 37.6, 4.4 Median, IQR: 38.2, 5.6 | 6355 |
| Albumin - maximum |  | Min-Max: 17 - 58  Mean, SD: 41, 4.1 Median, IQR: 42, 5 | 6355 |
| Albumin - minimum |  | Min-Max: 14 - 51  Mean, SD: 34, 6 Median, IQR: 35, 8 | 6355 |
| Albumin - standard deviation |  | Min-Max: 0 – 13.4  Mean, SD: 2.8, 1.6 Median, IQR: 2.4, 2 | 10930 |
| Albumin - latest value |  | Min-Max: 14, 57  Mean, SD: 37.7, 5.1 Median, IQR: 39, 6 | 6355 |
| Total Bilirubin - median | A bilirubin test measures the amount of bilirubin produced by the liver in your blood. It can reflect hepatic function, cancer disease status, or cancer treatment side effects.  The data is aggregated 1 year prior to each visit date. Each acronym denotes the following:   - med – Median value - mean – Mean - max – Maximum value - min – Minimum value - sd – Standard deviation - lat – Latest value | Min-Max: 3 - 450  Mean, SD: 12.4, 14.9 Median, IQR: 10, 5 | 7142 |
| Total Bilirubin - mean |  | Min-Max: 3 - 441  Mean, SD: 13.3, 16.2 Median, IQR: 10, 5.5 | 7142 |
| Total Bilirubin - maximum |  | Min-Max: 3 - 711  Mean, SD: 21.4, 38.6 Median, IQR: 13, 9 | 7142 |
| Total Bilirubin - minimum |  | Min-Max: 3 - 441  Mean, SD: 8.4, 8.3 Median, IQR: 7, 3 | 7142 |
| Total Bilirubin - standard deviation |  | Min-Max: 0 – 429.9  Mean, SD: 5.1, 13.2 Median, IQR: 2.5, 2.6 | 12279 |
| Total Bilirubin - latest value |  | Min-Max: 3 - 682  Mean, SD: 12.9, 18.3 Median, IQR: 10, 7 | 7142 |
| Creatinine Clearance - median | Creatinine clearance is the volume of blood plasma cleared of creatinine per unit time. It is a measurement of renal function.  Creatinine Clearance (ml/min/1.73 m²):  **2021 CKD-EPI Creatinine** = 142 x (Scr/*A*)*^B^* x 0.9938^AGE^x (1.012 if female)  The data is aggregated 1 year prior to each visit date. Each acronym denotes the following:   - med – Median value - mean – Mean - max – Maximum value - min – Minimum value - sd – Standard deviation - grad – Gradient of the robust regression plot - lat – Latest value | Min-Max: 3.7 - 166.4  Mean, SD: 91.9, 20 Median, IQR: 96.5, 20.6 | 3591 |
| Creatinine Clearance - mean |  | Min-Max: 3.9 - 165  Mean, SD: 91.3, 19.8 Median, IQR: 95.7, 21.2 | 3591 |
| Creatinine Clearance - maximum |  | Min-Max: 3.9 - 179.9  Mean, SD: 99.1, 19 Median, IQR: 101.5, 17.9 | 3591 |
| Creatinine Clearance - minimum |  | Min-Max: 1.8 - 147.1  Mean, SD: 81.5, 23.6 Median, IQR: 87.7, 31 | 3591 |
| Creatinine Clearance - standard deviation |  | Min-Max: 0 – 51.6  Mean, SD: 6.5, 5.2 Median, IQR: 5.2, 5.5 | 7986 |
| Creatinine Clearance - latest value |  | Min-Max: 3.2 - 179.9  Mean, SD: 91.3, 20.9 Median, IQR: 96, 22.3 | 3591 |
| Haemoglobin - median | Hemoglobin is measure of protein found in red blood cells that carries oxygen from the lungs to the body's tissues.  The data is aggregated 1 year prior to each visit date. Each acronym denotes the following:   - med – Median value - mean – Mean - max – Maximum value - min – Minimum value - sd – Standard deviation - lat – Latest value | Min-Max: 5.7-17.9  Mean, SD: 11.8, 1.8 Median, IQR: 11.8, 2.6 | 4859 |
| Haemoglobin - mean |  | Min-Max: 6 - 17.9  Mean, SD: 11.8, 1.7  Median, IQR: 11.8, 2.5 | 4859 |
| Haemoglobin - maximum |  | Min-Max: 6.3 - 20  Mean, SD: 13.2, 1.7 Median, IQR: 13.2, 2.2 | 4859 |
| Haemoglobin - minimum |  | Min-Max: 1 - 17.8  Mean, SD: 10.5, 2.3 Median, IQR: 10.5, 3.2 | 4859 |
| Haemoglobin - standard deviation |  | Min-Max: 0 - 6  Mean, SD: 1, 0.5 Median, IQR: 0.9, 0.7 | 8555 |
| Haemoglobin - latest value |  | Min-Max: 2.3 - 18.9  Mean, SD: 11.7, 1.9 Median, IQR: 11.8, 2.7 | 4859 |
| Platelet Count - median | Platelet count measured in a person’s blood, which assists with blood clots.  The data is aggregated 1 year prior to each visit date. Each acronym denotes the following:   - med – Median value - mean – Mean - max – Maximum value - min – Minimum value - sd – Standard deviation - lat – Latest value | Min-Max: 21 - 2593  Mean, SD: 274, 101.8 Median, IQR: 259, 115.5 | 4862 |
| Platelet Count - mean |  | Min-Max: 25.9 - 2284  Mean, SD: 279.3, 100.2 Median, IQR: 264, 115.9 | 4862 |
| Platelet Count - maximum |  | Min-Max: 26 - 3395  Mean, SD: 382.1, 161.6 Median, IQR: 350, 183 | 4862 |
| Platelet Count - minimum |  | Min-Max: 1 - 1301  Mean, SD: 201.9, 93 Median, IQR: 194, 115 | 4862 |
| Platelet Count - standard deviation |  | Min-Max: 0 - 1171.5  Mean, SD: 63.4, 46.4 Median, IQR: 53.9, 52.1 | 8573 |
| Platelet Count - latest value |  | Min-Max: 4 - 2227  Mean, SD: 267, 116.8 Median, IQR: 247, 130 | 4862 |
| White Blood Cell - median | Measure the number of white blood cells (leukocytes) in the blood.  The data is aggregated 1 year prior to each visit date. Each acronym denotes the following:   - med – Median value - mean – Mean - max – Maximum value - min – Minimum value - sd – Standard deviation - lat – Latest value | Min-Max: 0 - 69.6  Mean, SD: 7.1, 3.2 Median, IQR: 6.5, 3.3 | 4866 |
| White Blood Cell - mean |  | Min-Max: 0 - 67.9  Mean, SD: 7.5, 3.2 Median, IQR: 6.9, 3.4 | 4866 |
| White Blood Cell - maximum |  | Min-Max: 0 - 118.5  Mean, SD: 11.6, 7 Median, IQR: 9.9, 6.4 | 4866 |
| White Blood Cell - minimum |  | Min-Max: 0 - 60.5  Mean, SD: 5, 2.7 Median, IQR: 4.6, 3.1 | 4866 |
| White Blood Cell - standard deviation |  | Min-Max: 0 - 39.1  Mean, SD: 2.3, 1.9 Median, IQR: 1.9, 1.9 | 8576 |
| White Blood Cell - latest value |  | Min-Max: 0 - 111.8  Mean, SD: 7, 4.2 Median, IQR: 6.2, 3.6 | 4866 |
| Body Mass Index - median | Body Mass Index (BMI) is a person's weight in kilograms divided by the square of height in meters. BMI is a measure that uses your height and weight to work out if your weight is within healthy range. BMI change can reflect nutritional status and cancer anorexia/cachexia.  The data is aggregated 1 year prior to each visit date. Each acronym denotes the following:   - med – Median value - mean – Mean - max – Maximum value - min – Minimum value - sd – Standard deviation - lat – Latest value | Min-Max: 10.9 - 55.7  Mean, SD: 23, 4.2 Median, IQR: 22.6, 5.3 | 4704 |
| Body Mass Index - mean |  | Min-Max: 10.9 - 55.3  Mean, SD: 23, 4.2 Median, IQR: 22.6, 5.2 | 4704 |
| Body Mass Index - maximum |  | Min-Max: 10.9 - 57.2  Mean, SD: 23.9, 4.3 Median, IQR: 23.4, 5.4 | 4704 |
| Body Mass Index - minimum |  | Min-Max: 10.9 - 54.7  Mean, SD: 22.2, 4.2 Median, IQR: 21.8, 5.3 | 4704 |
| Body Mass Index - standard deviation |  | Min-Max: 0 - 13.3  Mean, SD: 0.6, 0.5 Median, IQR: 0.5, 0.5 | 10551 |
| Body Mass Index - latest value |  | Min-Max: 10.9 - 57.2  Mean, SD: 22.9, 4.3 Median, IQR: 22.5, 5.4 | 4704 |
| Neutrophil-Lymphocyte Ratio - median | Neutrophil-to-lymphocyte ratio (NL) is a parameter to assess the inflammatory status of a subject. It is a strong prognostic factor in several types of cancers and can be used as a predictor or marker of inflammatory or infectious pathologies.  The data is aggregated 1 year prior to each visit date. Each acronym denotes the following:   - med – Median value - mean – Mean - max – Maximum value - min – Minimum value - sd – Standard deviation - lat – Latest value | Min-Max: 0.3 - 84.6  Mean, SD: 4, 3.5 Median, IQR: 3, 2.7 | 4888 |
| Neutrophil-Lymphocyte Ratio - mean |  | Min-Max: 0.3 - 125.1  Mean, SD: 4.9, 4.2 Median, IQR: 3.7, 3.6 | 4888 |
| Neutrophil-Lymphocyte Ratio - maximum |  | Min-Max: 0.5 - 891.7  Mean, SD: 12.2, 18 Median, IQR: 7.1, 11.1 | 4890 |
| Neutrophil-Lymphocyte Ratio - minimum |  | Min-Max: 0 - 64.4  Mean, SD: 2.2, 2 Median, IQR: 1.7, 1.5 | 4888 |
| Neutrophil-Lymphocyte Ratio - standard deviation |  | Min-Max: 0 - 149.2  Mean, SD: 3.4, 5 Median, IQR: 1.9, 3.4 | 8596 |
| Neutrophil-Lymphocyte Ratio - latest value |  | Min-Max: 0.1 - 273  Mean, SD: 4.6, 6.3 Median, IQR: 2.9, 3.1 | 4888 |
| Albumin-Globulin Ratio - median | An AG ratio test measures albumin and globulin, the two main proteins in your blood. This blood test is used to monitor your nutritional status, immune function, and overall health. Its high expression level is found to be associated with longer survival time in cancer patients.  The data is aggregated 1 year prior to each visit date. Each acronym denotes the following:   - med – Median value - mean – Mean - max – Maximum value - min – Minimum value - sd – Standard deviation - lat – Latest value | Min-Max: 0.3 - 4.4  Mean, SD: 1.3, 0.3 Median, IQR: 1.3, 0.4 | 7240 |
| Albumin-Globulin Ratio - mean |  | Min-Max: -0.2 - 4.2  Mean, SD: 1.3, 0.3 Median, IQR: 1.3, 0.3 | 7240 |
| Albumin-Globulin Ratio - maximum |  | Min-Max: 0.3 - 8.2  Mean, SD: 1.5, 0.4 Median, IQR: 1.5, 0.4 | 7240 |
| Albumin-Globulin Ratio - minimum |  | Min-Max: -8.5 - 2.5  Mean, SD: 1.1, 0.3 Median, IQR: 1.2, 0.4 | 7240 |
| Albumin-Globulin Ratio - standard deviation |  | Min-Max: 0 - 3.1  Mean, SD: 0.1, 0.1 Median, IQR: 0.1, 0.1 | 12297 |
| Albumin-Globulin Ratio - latest value |  | Min-Max: -8.5 - 2.8  Mean, SD: 1.3, 0.3 Median, IQR: 1.3, 0.4 | 7240 |
| Unique number of cancer drugs given | The count of the number of unique drugs prescribed to a patient 14 days before diagnosis date to the current visit date. We use this as a s surrogate for change in cancer treatment line as this tends to portend poorer prognosis. | Min-Max: 0 - 12  Mean, SD: 2, 1.6 Median, IQR: 2, 2 | 0 |
| Alkylating drugs (L01A) | The number of times drugs L01 & L02 drugs were prescribed to a patient 14 days before diagnosis date to the current visit date. | 2376 | 0 |
| Antimetabolites (L01B) |  | 20506 | 0 |
| Plant alkaloids cancer drug (L01C) |  | 12386 | 0 |
| Cytotoxic Antibiotics (L01D) |  | 3028 | 0 |
| Protein Kinase Inhibitors (L01E) |  | 7095 | 0 |
| Monoclonal Antibody drugs (L01F) |  | 9470 | 0 |
| Antineoplastic drugs (L01X) |  | 22541 | 0 |
| Antigrowth hormone drugs (L02A) |  | 6435 | 0 |
| Hormone antonganist drugs (L02B) |  | 7534 | 0 |
| Trial Drugs | The number of trial drugs that were prescribed to a patient 14 days before diagnosis date to the current visit date. | 1697 | 0 |
| Congestive Heart Failure (ICD10) | The Elixhauser Comorbidity Index is a method of categorizing comorbidities of patients based on the International Classification of Diseases (ICD) diagnosis codes. Comorbidity severity has consistently been found to have an adverse impact on cancer survival.  The count captures the total number of comorbidities present from the earliest records to the current date of visit. | 876 | 0 |
| Cardiac Arrhythmia (ICD10) |  | 4119 | 0 |
| Valvular Disease (ICD10) |  | 518 | 0 |
| Pulmonary Circulation Disorder (ICD10) |  | 1107 | 0 |
| Peripheral Vascular Disease (ICD10) |  | 596 | 0 |
| Hypertension Uncomplicated (ICD10) |  | 13464 | 0 |
| Hypertension Complicated (ICD10) |  | 207 | 0 |
| Paralysis (ICD10) |  | 616 | 0 |
| Neurological Disorders (ICD10) |  | 744 | 0 |
| Chronic Pulmonary Disease (ICD10) |  | 1462 | 0 |
| Diabetes without Chronic Complications (ICD10) |  | 3449 | 0 |
| Diabetes with Chronic Complications (ICD10) |  | 9307 | 0 |
| Hypothyroidism (ICD10) |  | 404 | 0 |
| Renal Failure (ICD10) |  | 3005 | 0 |
| Liver Disease (ICD10) |  | 2056 | 0 |
| Peptic Ulcer Disease (ICD10) |  | 501 | 0 |
| AIDS/HIV (ICD10) |  | 68 | 0 |
| Lymphoma (ICD10) |  | 552 | 0 |
| Metastatic Cancer (ICD10) |  | 30248 | 0 |
| Solid Tumor without Metastasis (ICD10) |  | 50972 | 0 |
| Rheumatoid Arthritis (ICD10) |  | 241 | 0 |
| Coagulopathy (ICD10) |  | 1824 | 0 |
| Obesity (ICD10) |  | 118 | 0 |
| Weight Loss (ICD10) |  | 1268 | 0 |
| Fluid and Electrolyte Disorders (ICD10) |  | 8779 | 0 |
| Blood Loss Anemia (ICD10) |  | 384 | 0 |
| Anemia Defficiency (ICD10) |  | 2315 | 0 |
| Alcohol Abuse (ICD10) |  | 326 | 0 |
| Drug Abuse (ICD10) |  | 41 | 0 |
| Psychoses (ICD10) |  | 39 | 0 |
| Depression (ICD10) |  | 391 | 0 |
| Outpatient visits in last 1 year | Number of outpatient visits 90, 180, or 365 days before the current visit date.  Rise in healthcare utilization can be a surrogate for increasing health concerns/needs and a poor prognostic factor | Min-Max: 0 - 51  Mean, SD: 9.9, 7.7 Median, IQR: 8, 10 | 0 |
| Outpatient visits in last 6 months |  | Min-Max: 0 - 40  Mean, SD: 6.5, 5.2 Median, IQR: 5, 7 | 0 |
| Outpatient visits in last 3 months |  | Min-Max: 0 - 32  Mean, SD: 3.9, 3.4 Median, IQR: 3, 4 | 0 |
| Emergency Department visits in last 1 year | Number of emergency department visits 90, 180, or 365 days before the current visit date.  Rise in healthcare utilization can be a surrogate for increasing health concerns/needs and a poor prognostic factor | Min-Max: 0 - 13  Mean, SD: 0.7, 1.1 Median, IQR: 0, 1 | 0 |
| Emergency Department visits in last 6 months |  | Min-Max: 0 - 11  Mean, SD: 0.5, 0.8 Median, IQR: 0, 1 | 0 |
| Emergency Department visits in last 3 months |  | Min-Max: 0 - 7  Mean, SD: 0.3, 0.6 Median, IQR: 0, 0 | 0 |
| Inpatient Admission in last 1 year | Number of hospital inpatient visits 90, 180, or 365 days before the current visit date.  Rise in healthcare utilization can be a surrogate for increasing health concerns/needs and a poor prognostic factor | Min-Max: 0 - 19  Mean, SD: 1.1, 1.4 Median, IQR: 1, 2 | 0 |
| Inpatient Admission in last 6 months |  | Min-Max: 0 - 16  Mean, SD: 0.8, 1.1 Median, IQR: 0, 1 | 0 |
| Inpatient Admission in last 3 months |  | Min-Max: 0 - 11  Mean, SD: 0.5, 0.8 Median, IQR: 0, 1 | 0 |
| Age at prediction point | Patient’s age at the point of visit | Min-Max: 18 - 104  Mean, SD: 65.3, 10.9 Median, IQR: 66, 15 | 0 |
| 365-days mortality | Whether a patient dies within 1 year after a visit date. 1 if the patient dies, 0 if otherwise. | 0: 35389  1: 17149 | 0 |

**Table S3. Confusion Matrix for XGBoost**

|  |  | **Predicted** | |
| --- | --- | --- | --- |
|  |  | **0** | **1** |
| **Actual** | **0** | 6989 | 1673 |
|  | **1** | 1192 | 3268 |

**Table S4. Model parameters for XGBoost**

| **Hyperparameter** | **Grid search range** |
| --- | --- |
| Learning rate (eta) | 0.01 |
| Objective | binary:logistic |
| Base score | 0.32 |
| Evaluation metric | aucpr |
| Lambda | 50 |
| Gamma | 5 |
| Number of boosting iterations. | 5000 |
| Subsample | 0.5 |
| Scale position weight | 2.1 |
| Early stopping rounds | 20 |
| **Default Parameters** | |
| Colsample by tree | 1.0 |
| Alpha | 0 |
| Maximum depth | 6.0 |
| Minimum child weight | 1.0 |
| Max delta step | 0 |
